# Detection and Confirmation of Multiple Human Targets Using Pixel-Wise Code Aperture Measurements

**DOI:** 10.3390/jimaging6060040

**Published:** 2020-05-29

**Authors:** Chiman Kwan, David Gribben, Akshay Rangamani, Trac Tran, Jack Zhang, Ralph Etienne-Cummings

**Affiliations:** 1 Applied Research LLC, Rockville, MD 20850, USA; david.gribben00@gmail.com; 2 Electrical and Computer Engineering Dept., Johns Hopkins University, Baltimore, MD 21218, USA; arangam1@jhu.edu (A.R.); trac@jhu.edu (T.T.); retienne@jhu.edu (R.E.-C.); 3 Picower Institute for Learning and Memory, MIT, Cambridge, MA 02138, USA; jzhang1988@gmail.com

**Keywords:** detection, classification, YOLO, ResNet, pixel-wise code aperture, compressive measurement

## Abstract

Compressive video measurements can save bandwidth and data storage. However, conventional approaches to target detection require the compressive measurements to be reconstructed before any detectors are applied. This is not only time consuming but also may lose information in the reconstruction process. In this paper, we summarized the application of a recent approach to vehicle detection and classification directly in the compressive measurement domain to human targets. The raw videos were collected using a pixel-wise code exposure (PCE) camera, which condensed multiple frames into one frame. A combination of two deep learning-based algorithms (you only look once (YOLO) and residual network (ResNet)) was used for detection and confirmation. Optical and mid-wave infrared (MWIR) videos from a well-known database (SENSIAC) were used in our experiments. Extensive experiments demonstrated that the proposed framework was feasible for target detection up to 1500 m, but target confirmation needs more research.

## 1. Introduction

Compressive measurements [1] can be usually obtained by multiplying a Gaussian random matrix with the original vectorized image. Each measurement is a scalar, and many measurements are collected. Conventional detectors require the time-consuming reconstruction of the image scene first in order to provide accurate detection. Therefore, performing conventional target detection directly in the compressive measurement domain without a severe loss in the accuracy cannot be accomplished [2,3,4,5].

Pixel subsampling is a special case of compressive sensing because the Gaussian random matrix is a diagonal matrix with zeros in the off-diagonal entries. Detection and classification schemes have been proposed to directly utilize the pixel subsampling measures. Good results have been obtained in [2,3,4,5,6,7,8] as compared to some conventional algorithms.

Recently, a pixel-wise code exposure (PCE) camera was proposed [9]. The PCE camera is based on a special form of compressive sensing where multiple frames are compressed into a coded frame. To carry out target detection, it is necessary to reconstruct the original frames from the coded frames [9] using sparsity-based algorithms (L_1_ [10] or L_0_ [11,12,13]). It is well-known that it is computationally expensive to reconstruct the original frames. Consequently, real-time applications cannot be achieved by using PCE. Moreover, the reconstruction process normally loses information [14]. For practical and real-time applications, it is important to carry out target detection and classification directly using compressive measurements. In one paper [15] related to target tracking, the approach appeared to be using compressive measurements. However, original video frames were actually used. Other compressive measurement-based algorithms [16,17,18,19,20,21,22] assume the targets are already centered, which may not be realistic because targets can be anywhere in the image, and compressive measurements using the Gaussian random matrix lose the target location information. 

There are several publications written by us that have shown that PCE has also achieved good detection and classification results for vehicles [23,24,25,26] in optical and infrared videos. In this paper, we focused on the human target detection and classification approach using PCE measurements. Human targets are hard to detect and classify due to small bounding box size. Our scheme consisted of two steps. First, you only look once (YOLO) [27] was used for target detection. Other deep learning-based detectors, such as faster region-based convolutional neural network (R-CNN) [28] or single shot detector (SSD) [29], could be used. We adopted YOLO because it was compatible with our hardware. The training of the YOLO detector required image frames with known bounding boxes for the target locations. Due to a very limited number of video frames for training, the performance of YOLO for target classification was not good even though YOLO had a built-in classifier. As a result, we adopted the residual network (ResNet) [30] for target confirmation in the second step of our proposed approach. ResNet was chosen simply because a customized training via data augmentation could be easily done from the limited video frames. Low-quality videos in the SENSIAC database [31] were used to demonstrate our proposed approach. The detection and target confirmation results were good up to certain ranges.

In our experiments, we used the SENSIAC database in which there were two different sets of videos collected at different ranges. One set contained human subjects walking at a slow pace or gait, and this set was used for training. Based on the videos, a slow pace meant the humans were walking at normal speed. The other set had fast-moving (pace) human targets, and this set was used for testing. Based on the videos, the fast pace meant the humans were walking faster but not as fast as jogging. We trained the models for different types of cameras and different missing rates. For instance, for mid-wave infrared (MWIR) videos with a 50% missing rate, we trained one model that encompassed three ranges: 500 m, 1000 m, and 1500 m. This would reduce the number of trained models. We did not include conventional tracker results in this paper because, in our past studies [23,24,25,26], we observed that conventional trackers [32,33] failed in most cases.

Our contributions were as follows:

- Although the proposed scheme was not new and had been used by us for some other applications, we were the first ones to apply the PCE measurements to human target detection and confirmation. The SENSIAC database is very challenging in that the human targets are so small and hence difficult to detect and classify. To the best of our knowledge, we are not aware of any papers that deal with human target detection and confirmation by using that database.
- We demonstrated that human target detection and confirmation could indeed be done using coded aperture compressive measurements for long-range low-quality optical and MWIR videos.

The remainder of this paper is organized as follows. In Section 2, we have described some background materials, including the PCE camera, YOLO, and ResNet. In Section 3, we have first summarized the detection and confirmation results using SENSIAC optical videos. We have then presented the results for MWIR videos. Finally, we have included some remarks for future research in Section 4.

## 2. Methods and Data

### 2.1. PCE Imaging and Coded Aperture

**Figure 1:**
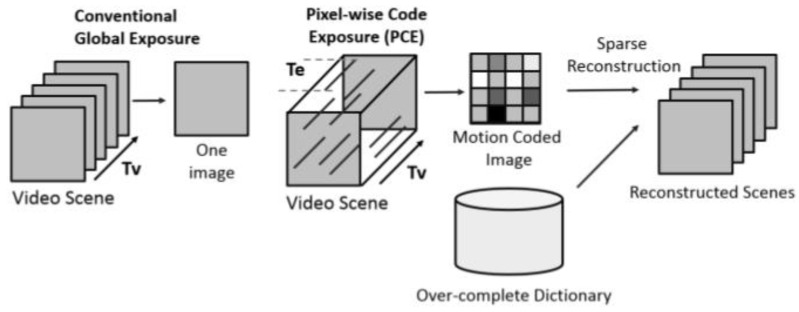
Conventional camera (left) vs. pixel-wise code exposure (PCE) camera (right) [9].

In this paper, we employed a compressive sensing scheme based on PCE or also known as coded aperture (CA) video frames, as described in [9]. Figure 1 shows a conventional camera and a PCE camera. One key feature in the PCE camera is that the pixels are activated randomly, and fixed temporal exposure duration is deployed for each pixel. There are several differences between conventional and PCE cameras. First, conventional cameras normally have fixed frame rates (15 or 30 frames per second). In contrast, a PCE camera compresses multiple frames into a motion coded image over a fixed period of time (Tv). For example, 30 conventional frames can be compressed into a single motion coded frame. Consequently, significant data compression can be achieved. Second, the PCE camera can apply different exposure times for different pixel locations based on lighting conditions. That is, more exposure times can be given to low lighting regions, and short exposure can be used for strong light areas. Consequently, a high dynamic range can be attained. Moreover, power consumption can be controlled via sampling rates in the data acquisition hardware. As shown in Figure 1, we also included one conventional approach to using the motion coded images by applying sparse reconstruction to reconstruct the original frames from the motion coded images. However, this process was time-consuming and hence not suitable for practical applications that demand near real-time operations. 

The coded aperture image $Y\in R^{M\times N}$ was obtained by

(1) $$Y(m,n)=\sum_{t=1}^{T}S(m,n,t)\cdot X(m,n,t)$$

where $X\in R^{M\times N\times T}$ denotes a sequence of *T* video frames, and each frame has a size of *M* × *N*; $S\in R^{M\times N\times T}$ denotes the sensing data cube in which the value of S(*m,n,t*) is 1 for frames *t* ∈ [*t_start_*, *t_end_*] and 0 otherwise. [*t_start_*, *t_end_*] denotes the start and end frame numbers for a particular pixel. In our experiments, the compression was done every 5 frames. Therefore, starting at the beginning of each video, the starting frame *t_start_* would be 0, and the final frame *t_end_* would be 4. From there, this process would continue until the final frame. For instance, let the final frame be 1799 (assuming an 1800 frame video and an index starting at 0), the last coded aperture frame would have a *t_start_* of 1795, and *t_end_* would be 1799. If the number of frames in the video was not divisible by 5, then the final condensed image would then only have whatever remainder was left after the full compression of 5 frames.

The video frames in $S\in R^{M\times N\times T}$ could be reconstructed via sparse representation methods (*L_1_* or *L_0_*). Details could be found in [9]. 

**Figure 2:**
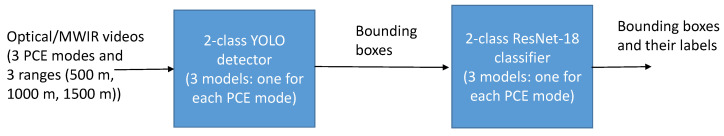
The relationship between you only look once (YOLO) and residual network (ResNet) for human target detection and confirmation.

In our proposed scheme shown in Figure 2, we did not perform sparse reconstruction on PCE images, as we directly performed detection and target confirmation using PCE images. There are several challenges in utilizing raw PCE measurements. First, if the exposure times are long, moving targets may be smeared, and this will affect the detection and confirmation quality. Second, not all pixels are activated during the data collection process, and hence there are missing pixels in the raw measurements. Third, there are much fewer frames in the raw PCE video due to the compression process in PCE. Consequently, training data will be limited, and models will be harder to train. In our proposed detection and classification scheme, we first applied YOLO to the PCE measurements to detect the human targets. The bounding boxes of those targets would be fed into ResNet for target confirmation. The outputs would be the bounding boxes and the labels of each bounding box.

Our team has been working on another project related to self-driving cars. One key advantage of code aperture camera is that it has a high dynamic range, low power consumption, low data storage, and low bandwidth usage. Consequently, potential applications may include wide-area surveillance using unmanned air vehicles, security monitoring, self-driving car safety enhancement, etc.

In this paper, we focused on simulating PCE measurements. Our goal was to demonstrate that detecting and classifying moving human targets is feasible. Three diverse sensing models: PCE/CA Full, PCE/CA 50%, and PCE/CA 25% were carried out in our experiments. Full means that there were no missing pixels. We also denoted this case as a 0% missing case. PCE 50% and PCE 25% cases mean that 50% and 75% of the pixels in each frame were also missing in the PCE measurements, respectively. More details could also be found in [23,24,25,26]. 

### 2.2. YOLO

YOLO tracker [27] is faster and comparable in accuracy to faster R-CNN [28]. In contrast to typical detectors that look at multiple locations in an image and return the highest scoring regions as detections, YOLO, as its namesake explains, looks at the entire image to make determinations on detections, giving each scoring global context. This method makes the prediction extremely fast, up to one thousand times faster than an R-CNN. It also works well with our current hardware. It is easy to install, requiring only two steps and few prerequisites. This differs greatly from many other detectors that require a very specific set of prerequisites to run a Caffe-based system. YOLO works without the need for a GPU but, if initialized in the configuration file, easily compiles with the compute unified device architecture (CUDA), which is the NVIDIA toolkit, when constructing the build. YOLO also has a built-in classification module. However, the classification accuracy using YOLO is poor according to our past studies [23,24,25,26]. While the poor accuracy may be due to a lack of training data, the created pipeline that feeds input data into both YOLO and the classifier is already set up to provide more training data to ResNet and is, therefore, more effective at providing results.

One key advantage of YOLO is its speed, as it can predict multiple bounding boxes per grid cell of size 7 x 7. For the optimization process during training, YOLO uses a sum-squared error between the predictions and the ground truth to calculate the loss. The loss function comprises the classification loss, the localization loss (errors between the predicted boundary box and the ground truth), and the confidence loss. More details can be found in [27].

YOLO has its own starter model—Darknet-53—that can be used as a base to further train a given dataset. It contains, as the name would suggest, 53 convolutional layers. It is constructed in a way to optimize speed while also competing with larger convolutional networks. Examples used for comparison are both ResNet-101 and ResNet-152. Darknet-53 is faster and more accurate than these two models for detection. There is no specific mention of classification, but as we have discovered, ResNet model ResNet-18 performs better classification accuracy than Darknet-53. 

In the training of YOLO, we trained three separate models for each missing rate. In several tests, we found the missing rate to be the dominant factor in testing accuracy rather than the distance from the target. A method used to increase accurate detections of human targets was having a two-class model rather than a one-class model. The hypothesis was that, due to the small number of human bounding boxes, it was necessary to increase the number of overall bounding box images to help create a well-trained model that could distinguish between the target and background. The best method to do that without providing any data augmentation was to include a background class in training. An added benefit to this two-class model was that the Darknet-53 author instructions for maximum batches were to run the training for 2000 times the number of classes. By increasing the number of classes, we were also able to increase the maximum batches to further help create a well-trained model.

There may be other deep learning-based detectors, such as SSD, in the literature. We tried to use SSD. After some investigations, we observed that it was very difficult to custom train it. In any event, our key objective was to demonstrate human target detection and confirmation using PCE measurements. Any relevant detectors could be used.

### 2.3. ResNet

We used the ResNet-18 model, which is an 18-layer convolutional neural network (CNN). One key advantage of ResNet is that it can avoid performance saturation and/or degradation when training deeper layers. This is done by introducing an identity shortcut connection in the model, which skips one or more layers and learns the residual mapping of the layer rather than the original mapping. The main reason for choosing the 18-layer version of ResNet is that the larger networks become increasingly difficult to retrain. The 18-layer model is a good balance of being large enough to have good accuracy but small enough to be malleable for retraining.

ResNet uses cross-entropy as the loss function during training. Cross entropy has been widely used in the training of many deep learning models. In [30], the optimization details and variants of ResNet are discussed.

ResNet was used in addition to YOLO, not only because it had consistently higher classification accuracies, as was observed in past investigations but also to act as a second layer of classification to catch anything YOLO might have missed. Due to the way YOLO was trained, having two classes, it was specifically designed to over detect when finding human targets. This was when any detection was passed from ResNet to YOLO. It was then ResNet’s job to act as a second layer of safeguard in order to remove any false positives or background images that were detected in the previous step. 

When we trained the ResNet classifier, we used the videos with a slow gait. The targets cropped from the videos were also augmented with scaling (enlarge by 50% and shrink by 50%), rotation by every 10 degrees, and illumination changes (brighten by 50% and darken by 50%). This led to an overall increase in training data from approximately 10,000 frames to 1,500,000 frames. Depending on the number of subjects in a given video, that number almost tripled as a majority of the frames had 3 subjects of intersecting paths.

We trained the ResNet based on missing rates. That is, for each missing rate, we had one trained ResNet model regardless of the ranges of the videos.

### 2.4. Data

In this paper, the well-known SENSIAC dataset was used. This dataset was compiled by the US army night vision and electronic sensors directorate (NVESD) in an effort to aid the development of algorithms related to automated target recognition. In total, it contained over 300 GB of MWIR and visible imagery. Although we were currently focusing on human subjects, the full database included foreign military and civilian vehicles. 

The camera that collected MWIR imagery was the L3 Cincinnati Electronics Night Conqueror, which was then paired with the great river frame grabber to extract the data. This camera had a 640 by 480-pixel indium antimonide focal plane array and was outfitted with a 300 mm lens to produce a 3.4 by 2.6 field of view (FOV). The optical camera was manufactured by Illunis and was outfitted with a Nikon zoom lens to produce a 3.4 Horizontal Field of View (HFOV) while locked in position. Frames were then collected using the Coreco frame grabber. 

For each camera, the captured imagery covered 500 to 3000-meter ranges in 500-meter increments. The objective of the dataset was to obtain 72 aspect angles of each target, which was done by having a set circular path for the various subjects to travel along for each distance. Therefore, the subject would rotate a full 360 degrees, and each part of the subject would be in view throughout the trials. The set path also made for consistent data collection. It was clear from the sheer size of the vehicular portion of the dataset that the focus was on those subjects rather than the human targets. However, there was still a decent amount of human subject data collected for all distances. It should be noted that in the user guide for the SENSIAC data, there is a comment that on the day of data collection, a project head noticed the large variations in land height and surrounding sight obstructions for the 500-meter range and discontinued data collection for that distance. As a result, the only images in the dataset at the 500-meter range were for human targets. 

Each distance for the human trials had both a slow gait and fast gait recorded. As a result, for the human subset of the SENSIAC dataset, there were a total of 24 videos across all variations of recording method and distance. However, videos in ranges farther than 1500 m were not used because the human targets were too small to achieve any credible detection or classification results. Each model was trained on a relatively small dataset of 6 videos for training and 6 for testing, 3 for each category in MWIR and optical. The 6 training videos had the human subjects walking at the slow gait mentioned above, while the 6 testing videos were of the faster gait. The total number of unique training images reached a little over 10,000 frames. With data augmentation, that number increased to around 1,500,000 images. The average size of those bounding box images was around 19 pixels by 10 pixels with the 1500 meter bounding boxes reaching a total area of 120 total pixels. A meager 0.039% of the 307,200 pixels of the 640 by 480 image.

### 2.5. Performance Metrics

For detection, we used the following metrics:

- Center location error (CLE): It is the error between the center of the bounding box and the ground-truth bounding box. Smaller means better. CLE is calculated by measuring the distance between the ground truth center location ($C_{x,gt},C_{y,gt}$) and the detected center location ($C_{x,est},C_{y,est}$). Mathematically, CLE is given by:
  (2) $$CLE=\sqrt{{\left(C_{x,est}-C_{x,gt}\right)}^{2}+{\left(C_{y,est}-C_{y,gt}\right)}^{2}}$$
- Distance precision (DP): It is the percentage of frames where the centroids of detected bounding boxes are within 10 pixels of the centroid of ground-truth bounding boxes. Close to 1 or 100% indicates good results.
- Estimates in ground truth (EinGT): It is the percentage of the frames where the centroids of the detected bounding boxes are inside the ground-truth bounding boxes. It depends on the size of the bounding box and is simply a less strict version of the DP metric. Close to 1 or 100% indicates good results.
- Mean area precision (mAP): mAP calculates the amount of area overlap for the estimated and ground truth bounding boxes compared to the total area of the two bounding boxes and returns the result as a value between 0 and 1, with 1 being the perfect overlap. The mAP being used can be computed as
  (3) $$mAP=\frac{Area\;of\;Intersection}{Area\;of\;Union}$$
  As shown in Equation (3), mAP is calculated by taking the area of intersection of the ground truth bounding box and the estimated bounding box, then dividing that area by the union of those two areas.
- The number of frames with detection: This is the total number of frames that have detection.

For classification, we used confusion matrix and classification accuracy as performance metrics.

## 3. Detection and Confirmation Results Using PCE Measurements

We investigated optical and MWIR videos using PCE measurements. Although there were videos collected up to 3000 m, we focused on videos up to 1500 m because human targets were too tiny beyond 1500 m.

### 3.1. Results on Optical Videos

There were three missing cases in PCE measurements: 0%, 50%, and 75%. Moreover, in the PCE measurements, five frames were compressed into one frame.

#### 3.1.1. Detection Results

**Figure 3:**
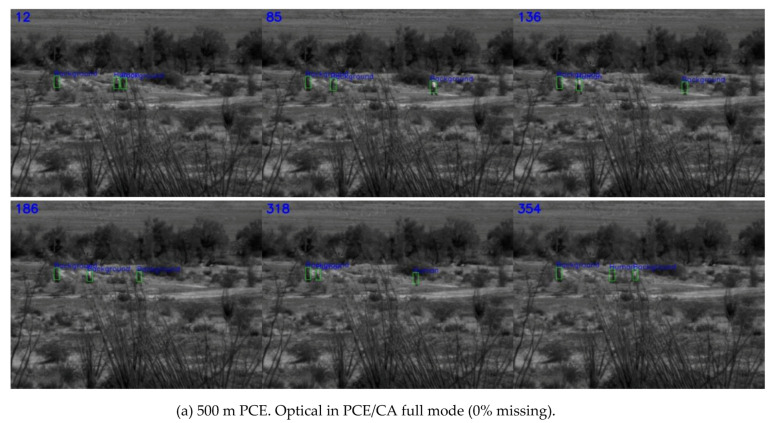

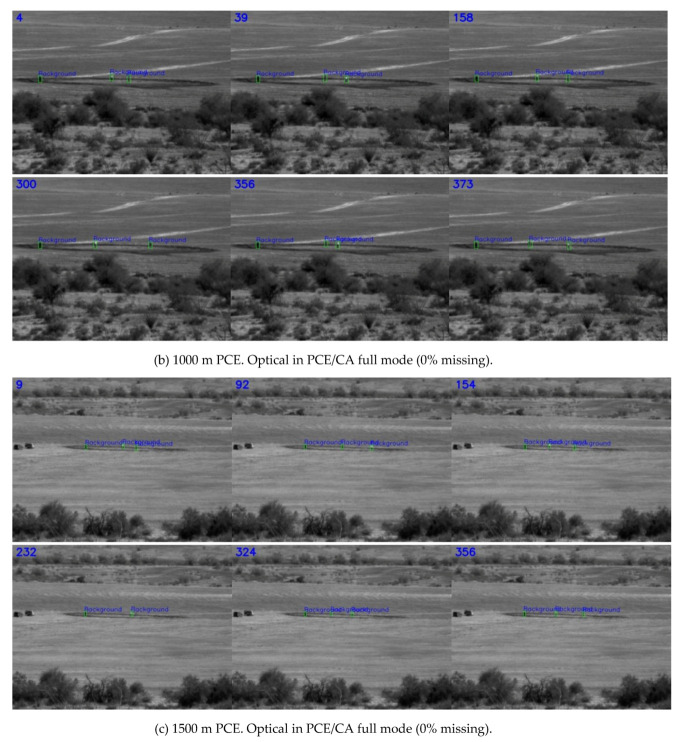
Detection results using optical coded aperture in PCE/CA full mode (0% missing). CA, coded aperture.

**Figure 4:**
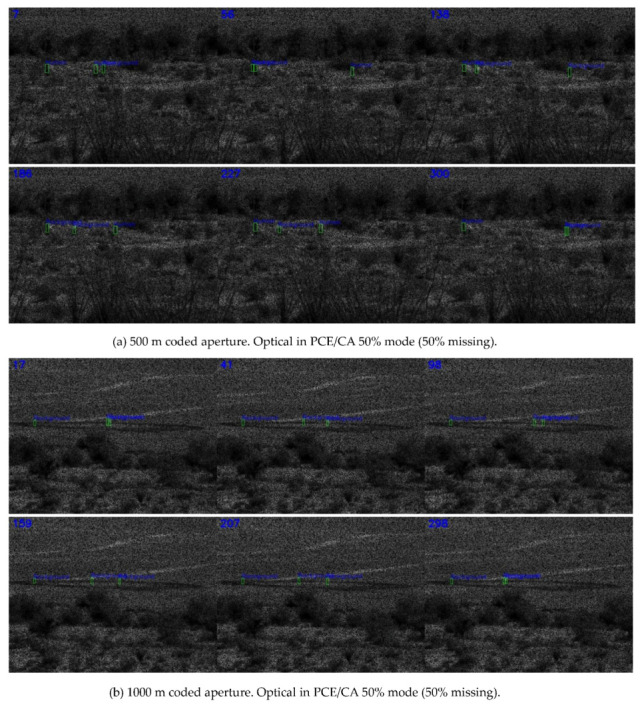

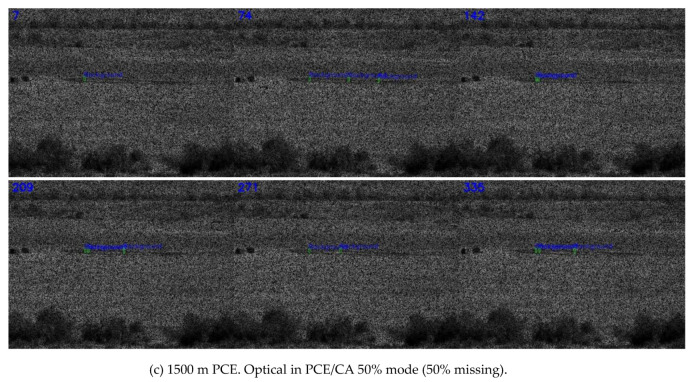
Detection results using optical coded aperture in PCE/CA 50% mode (50% missing).

**Figure 5:**
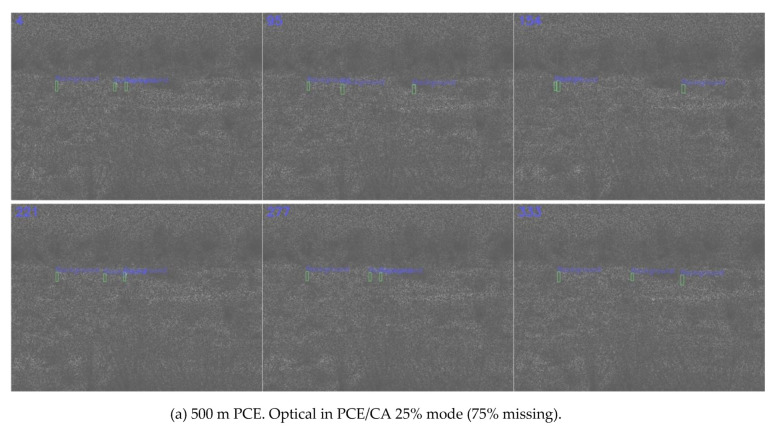

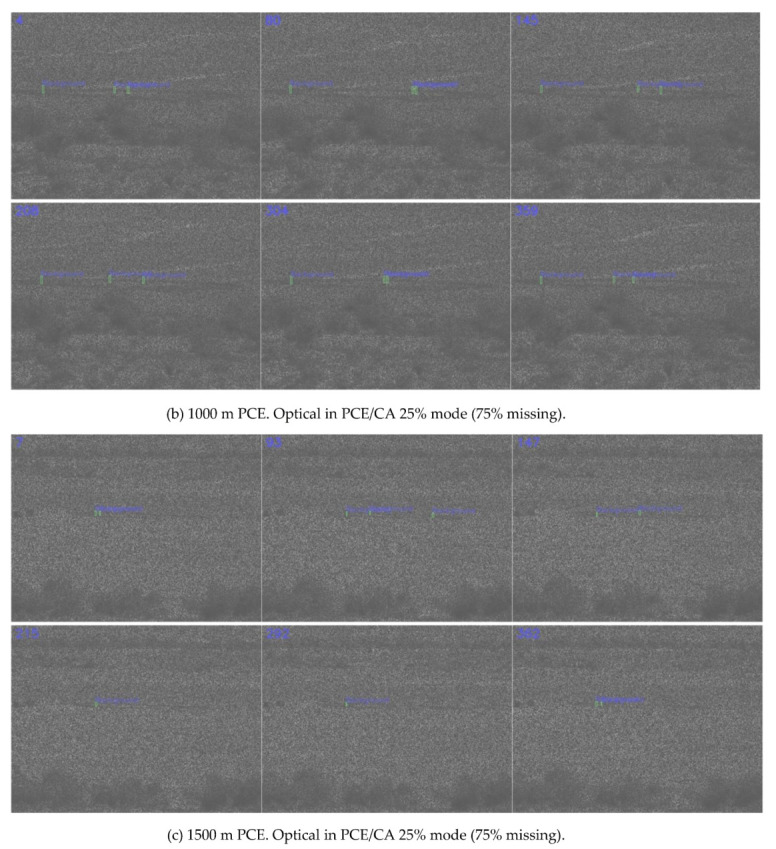
Detection results using optical coded aperture in PCE/CA 25% mode (75% missing).

Three separate YOLO (Darknet-53) detection models were trained, as mentioned previously, using slow pace videos at three ranges (500 m, 1000 m, and 1500 m) at 0%, 50%, and 75% missing cases, respectively. That is, we had three distinct models, specifically for 0%, 50%, and 75% missing cases. From Table 1, the general trend was that higher missing rates had lower performance metrics, but not always. A short-range should have better results. However, at 500 m range, there was a lot of background and foreground clutter, which affected the detection performance. Consequently, the metrics at 500 m were not the best. As was mentioned in the data portion, the 500-meter case was not considered a reliable distance for signature collection, explaining the unexpected result of decreased performance. The snapshots in Figure 3, Figure 4 and Figure 5 corroborated the above argument. It could be seen that the longer ranges had less clutter. From Figure 3a, which shows the 500 m range case, there are six frames in which the frame numbers are shown at the top left corner. It could be seen that the green bounding boxes were tightly around the three human targets despite the presence of heavy clutter in the background. Similarly, we could see that the bounding boxes were also tightly around the human targets at longer ranges of 1000 m and 1500 m, as shown in Figure 3b,c, respectively.

Figure 4a shows the detection results of PCE measurements with 50% missing pixels. The frames looked darker because 50% of the pixels were missing. One could see that the green bounding boxes could still be seen to be tightly around most of the human targets. Similarly, at longer ranges of 1000 m and 1500 m, the detection results, shown in Figure 4b,c, were also adequate.

Figure 5a shows the detection results with 75% missing pixels. The frames became dark due to the lack of pixels. However, the green bounding boxes could still be seen to be around the human targets in most frames.

#### 3.1.2. Human Target Confirmation Results

To assess the target confirmation performance of ResNet, we used confusion matrices.

Similar to the detection case, we had three separate classification models for 0%, 50%, and 75% missing cases that then classified detections for the three ranges of 500, 1000, and 1500 m. There were six confusion matrices. The classification results for 75% missing cases were poor (0% accuracy), and we did not include those results in order to save some space. As shown in Table 2, the 0% missing 500 m case was surprising. We repeated our experiments a few times and still got the same results. We thought there were two reasons. First, there was a lot of background clutter, as could be seen in Figure 3a. Another reason was that the testing videos contained fast pace targets, and there was more smear in the coded aperture frames due to motion. For longer ranges, the clutter appeared to have less impact (see Figure 3b,c). For 50% missing case at 500 m range, 50% of targets were classified as humans, and another 50% were classified as background. The results were understandable because the human targets were so small from a distance. For other cases, we observed that a lot of background boxes were wrongly classified as humans. In general, the classification results were not satisfactory for optical videos. 

### 3.2. Results on MWIR Videos

For each range, we had three missing rates (PCE modes) for the PCE measurements.

#### 3.2.1. Detection Results

**Figure 6:**
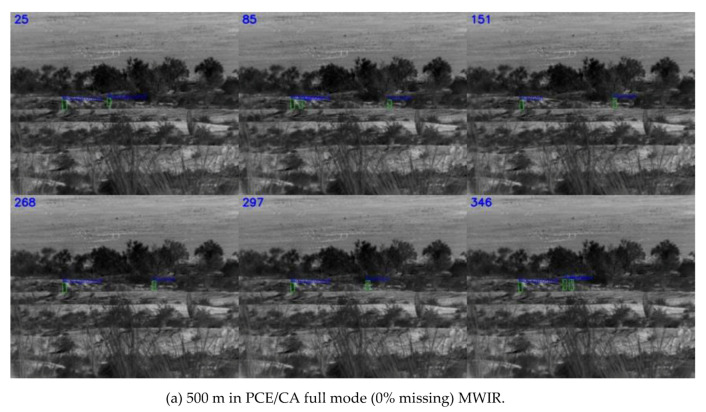

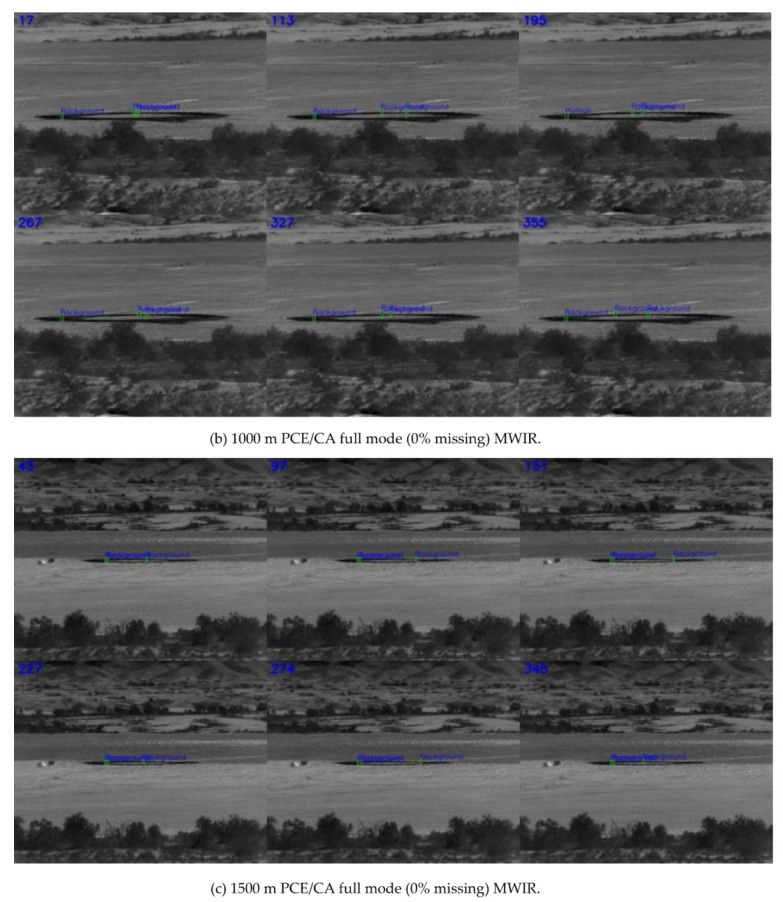
Detection results for MWIR in PCE/CA full mode (0% missing). MWIR, mid-wave infrared.

**Figure 7:**
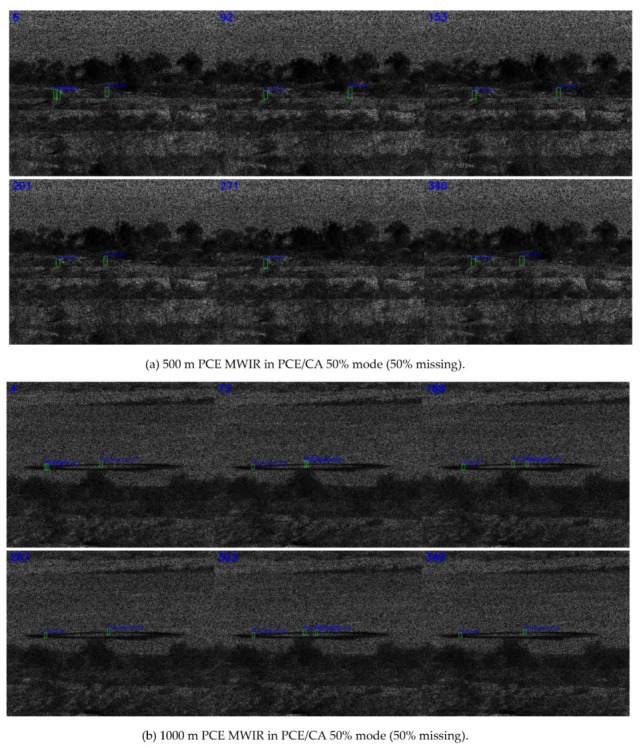

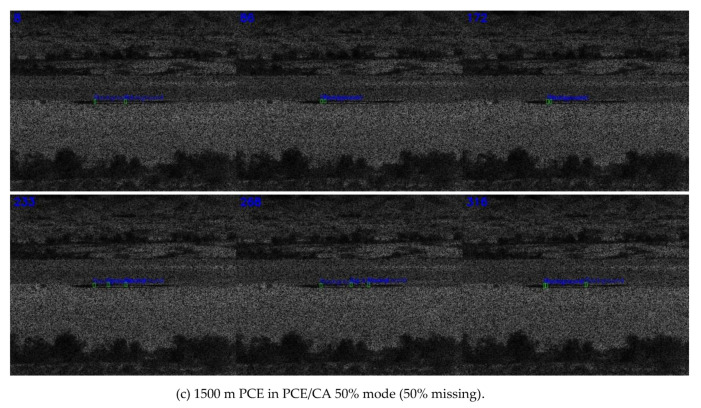
Detection results of MWIR PCE in PCE/CA 50% mode (50% missing).

**Figure 8:**
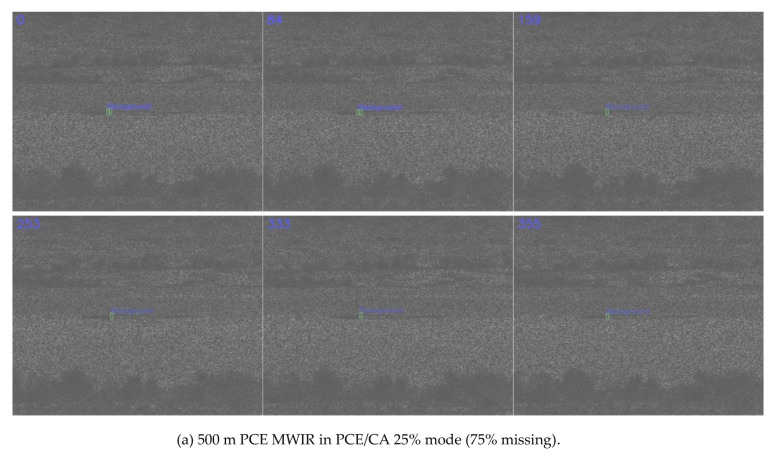

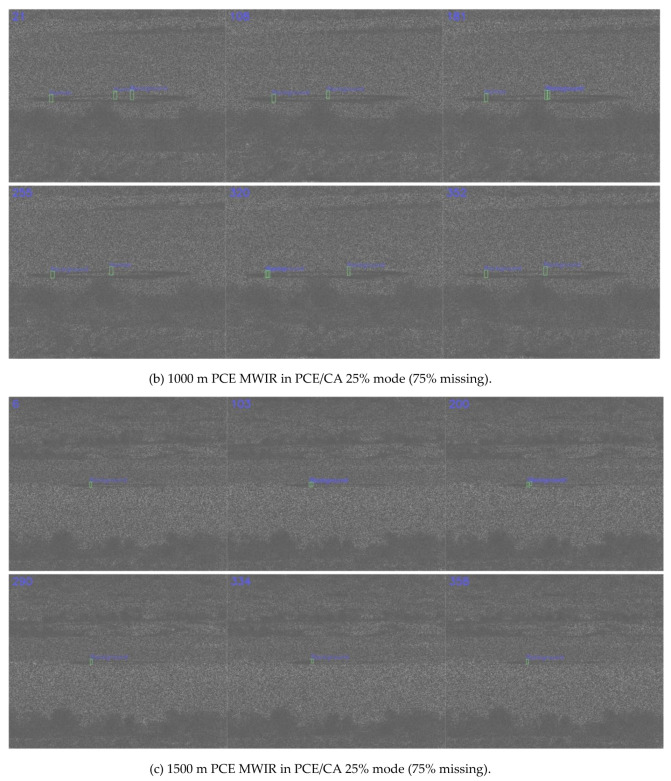
Detection results for MWIR PCE in PCE/CA 25% mode (75% missing).

Three separate detection models were trained using slow pace videos at three ranges (500 m, 1000 m, and 1500 m) at 0%, 50%, and 75% missing cases, respectively. That is, we had three distinct models, specifically for 0%, 50%, and 75% missing cases. For each missing rate, slow pace videos from three different ranges were used in the training process. The cropped targets were also augmented with different scaling, illumination, and orientations. Once trained, each model was applied to those fast pace videos at three different ranges and missing rates. Only detected targets were fed into the testing model in each video. As shown in Table 3, for the MWIR videos, the trends in the various metrics were, in general, correct. That is, shorter ranges had better performance, and lower missing rates had better performance. The lower percentage of detection in the 500 m range as compared to the 1000 m range was probably because there was more clutter in the background. Snapshots in Figure 6, Figure 7 and Figure 8 corroborated the above argument. 

Comparing Table 1 and Table 3, one could notice that the MWIR at 500 m range had relatively poor performance. This was probably because MWIR images might be more susceptible to background clutter, as those clutters were shrub and small trees that also emit heat.

#### 3.2.2. Target Confirmation Results

Table 4 summarizes the classification results using ResNet for MWIR videos. There were six confusion matrices. Three separate models were trained for the three missing cases: 0%, 50%, and 75%. Similar to the optical case, the 75% missing cases did not have good results (0% accuracy), and we did not include those results. For each missing rate, slow pace videos from three different ranges were used in the training process. The cropped targets were also augmented with different scaling, illumination, and orientations. Once trained, each model was applied to those fast pace videos at three different ranges. Only detected targets were fed into the testing model in each video. From Table 4, the results were not satisfactory. The ResNet could only classify human targets but could not correctly classify background bounding boxes. In short, the ResNet was not effective in differentiating human targets from the background. More research is needed. 

### 3.3. Discussion of the Experimental Results

#### 3.3.1. Detection Performance Comparison between Optical and MWIR Imagers

At PCE full (0% missing) and PCE half (50% missing) cases, the detection performances of optical and MWIR images were similar. At 75% missing cases, the MWIR imager had a slight edge over the optical imager. For instance, the mAP of MWIR at 75% missing and 500 m range case was 0.38, whereas the mAP of optical image in the same case was 0.36.

#### 3.3.2. Target Confirmation Performance Comparison between Optical and MWIR Imagers

At all ranges, the classification performances of optical and MWIR imagers were both poor. In the 500 m range, the MWIR performances in PCE 50% mode were slightly better than the optical counterparts. One explanation might be due to the fact that the MWIR imager was less susceptible to air turbulence. In some optical videos, the desert heat could create air turbulence, which could create slight distortions in images.

#### 3.3.3. Target Confirmation Accuracy of ResNet

Although the ResNet classifier had been proven to be more accurate than YOLO’s built-in classifier in our past studies, we would like to emphasize that the performance of ResNet was not good in our experiments. This was because the ranges were far and the human targets were small, even from a range of 500 m. One way to improve the classification performance was to investigate super-resolution methods. Another potential direction is to perform atmospheric turbulence compensation.

#### 3.3.4. Comparison with Conventional Reconstruction Methods

As mentioned in Section 1, conventional reconstruction-based methods are time-consuming and may also lose target information. Some researchers have looked into the target classification using compressive measurements. However, those methods in [16,17,18,19,20,21,22] require targets to be centered in the image, which is unrealistic in practical applications. In contrast, our approach does not require the targets to be centered. Actually, the targets can be anywhere in the image scene.

#### 3.3.5. Incorporation of Tracking into our Framework

The tracking problem is interesting and has been studied in recent years [34,35,36]. We plan to carry out some tracking investigations in the future.

## 4. Conclusions

Human targets are small and hard to track and classify at long ranges. In this paper, our goal was to investigate the performance of a compressive sensing approach to detecting and classifying multiple humans directly using compressive measurements without any image reconstruction. A deep learning approach combining YOLO and ResNet was used to achieve the above goal. Realistic optical and MWIR videos were used in our experiments. Our approach was modular and, hence, newer and more powerful deep learning models could be used when they are available. Multiple targets could be simultaneously detected. However, the effectiveness was only up to 500 m due to the small human target size. The classification results from the deep learning method of ResNet produced poor results. This was understandable because the target size was very small.

In the future, we plan to implement a real-time framework for target detection and classification using PCE measurements. Moreover, some super-resolution algorithms may be incorporated to enhance the image quality so that the tracking and classification can be further improved. 

## Figures and Tables

**Table 1:** Detection using optical videos. Bold numbers indicate the best results among the different ranges. Bold numbers indicate the best metric among all the ranges.

PCE/CA Full (0% Missing)					
	**CLE**	**DP@10**	**EinGT**	**mAP**	**% Detection**
500 m	3.03	**1.00**	0.98	0.38	95.01
1000 m	**1.45**	**1.00**	**1.00**	**0.69**	**97.95**
1500 m	1.78	**1.00**	0.94	0.49	95.81
**PCE/CA 50% (50% Missing)**					
	**CLE**	**DP@10**	**EinGT**	**mAP**	**% Detection**
500 m	**4.94**	**0.98**	**0.76**	**0.45**	94.39
1000 m	11.02	0.83	0.55	0.38	**96.52**
1500 m	10.57	0.64	0.22	0.14	66.31
**PCE/CA 25% (75% Missing)**					
	**CLE**	**DP@10**	**EinGT**	**mAP**	**% Detection**
500 m	**18.47**	**0.89**	**0.51**	**0.36**	96.88
1000 m	18.13	0.63	0.14	0.15	**99.38**
1500 m	20.14	0.42	0.12	0.07	69.43

**Table 2:** ResNet confirmation results for optical videos using PCE. There were six confusion matrices. GT stands for ground truth.

	PCE/CA Full (0 % Missing)		PCE/CA 50% (50% Missing)	
**Range: 500**	**GT: Human**	**GT: Background**	**GT: Human**	**GT: Background**
Classified: Human	0%	100%	87%	91%
Classified: Background	100%	0%	13%	9%
**Range: 1000**	**GT: Human**	**GT: Background**	**GT: Human**	**GT: Background**
Classified: Human	50%	50%	95.02%	94.66%
Classified: Background	50%	50%	4.98%	5.34%
**Range: 1500**	**GT: Human**	**GT: Background**	**GT: Human**	**GT: Background**
Classified: Human	87%	98%	99.88%	99.56%
Classified: Background	13%	2%	0.12%	0.44%

**Table 3:** MWIR PCE video detection results. Bold numbers indicate the best results among the different ranges. Bold numbers indicate the best metric among all the ranges.

PCE/CA Full (0% Missing)					
	**CLE**	**DP@10**	**EinGT**	**mAP**	**% Detection**
500 m	**2.48**	**1.00**	**0.96**	**0.52**	85.98
1000 m	2.25	1.00	0.80	0.52	96.84
1500 m	2.79	1.00	0.39	0.22	**99.72**
**PCE/CA 50% (50% Missing)**					
	**CLE**	**DP@10**	**EinGT**	**mAP**	**% Detection**
500 m	**3.70**	**0.98**	**0.92**	**0.44**	69.92
1000 m	7.42	0.85	0.64	0.38	**94.99**
1500 m	11.48	0.71	0.18	0.13	77.44
**PCE/CA 25% (75% Missing)**					
	**CLE**	**DP@10**	**EinGT**	**mAP**	**% Detection**
500 m	**10.41**	**0.95**	**0.91**	**0.38**	58.77
1000 m	13.43	0.66	0.43	0.25	**79.11**
1500 m	10.79	0.49	0.03	0.05	46.61

**Table 4:** ResNet classification results for MWIR videos in PCE mode.

	PCE/CA Full (0% Missing)		PCE/CA 50% (50% Missing)	
**Range: 500**	**GT: Human**	**GT: Background**	**GT: Human**	**GT: Background**
Classified: Human	94.32%	93.43%	99.11%	98.17%
Classified: Background	5.68%	6.57%	0.89%	1.83%
**Range: 1000**	**GT: Human**	**GT: Background**	**GT: Human**	**GT: Background**
Classified: Human	95.54%	93.54%	99.85%	100%
Classified: Background	4.46%	6.46%	0.15%	0%
**Range: 1500**	**GT: Human**	**GT: Background**	**GT: Human**	**GT: Background**
Classified: Human	92.26%	92.06%	100%	100%
Classified: Background	7.74%	7.94%	0	0
